# Clinical and functional characterization of a novel STUB1 mutation in a Chinese spinocerebellar ataxia 48 pedigree

**DOI:** 10.1186/s13023-024-03456-8

**Published:** 2024-12-20

**Authors:** Jiaqi Li, Wenyi Xie, Jian-Min Chen, Chun-Zuan Xu, Ya-Li Huang, Sheng Chen, Chang-Yun Liu, Ying-Qian Lu, Zhang-Yu Zou

**Affiliations:** 1 Department of Neurology, Fujian Medical University Union Hospital, Fuzhou, China; 2 Institute of Clinical Neurology, Fujian Medical University, Fuzhou, China

**Keywords:** SCA48, SCAR16, *STUB1* gene, CHIP, Ubiquitin

## Abstract

**Background:**

Spinocerebellar ataxias (SCAs) encompass a wide spectrum of inherited neurodegenerative diseases, primarily characterized by pathological changes in the cerebellum, spinal cord, and brainstem degeneration. Autosomal dominant spinocerebellar ataxia type 48 (SCA48) is a newly identified subtype of SCA, marked by early-onset ataxia and cognitive impairment, and is associated with mutations in the *STIP1 homology and U-box-containing protein 1* (*STUB1)* gene. The *STUB1* gene encodes the protein CHIP (C-terminus of HSC70-interacting protein) which functions as E3 ubiquitin ligase and is crucial to the development of neural systems.

**Results:**

Here, we reported a Chinese SCA48 family exhibited typical features and defined a novel missense mutation *STUB1* c.755A>C (CHIP p. Y252S) through whole-exome sequencing. The variant was interpreted as a variant of uncertain significance, so we conducted a series of experiments using minigene plasmids to evaluate the pathogenicity of the variant. We found that the variant *STUB1* c.755A>C caused a significant reduction of CHIP level and the loss function of ubiquitin ligase activity as the pathogenic *STUB1* mutations reported before. Besides, we also found that the CHIP p. Y252S could cause tau aggregation, which is considered to contribute to the progression of neurodegenerative disorders.

**Conclusions:**

We diagnose the SCA48 pedigree in China and highlight the role of decreased ubiquitination and increased tau aggregation in the pathogenesis of the novel *STUB1* c.755C>A mutation.

**Supplementary Information:**

The online version contains supplementary material available at 10.1186/s13023-024-03456-8.

## Background

Spinocerebellar ataxias (SCAs) represent a collection of inherited neurodegenerative disorders characterized by the gradual onset of symptoms such as gait ataxia and dysarthria [1]. To date, about 50 distinct forms of SCAs have been identified, each associated with mutations occurring in a diverse range of genes, and the mode of inheritance can also vary, including autosomal dominant inheritance, autosomal recessive inheritance, and X-linked inheritance [2, 3]. Expanded polyglutamine repeats (CAG, polyQ) account for the predominant forms of SCAs, with other noncoding repeat expansions and substitutions also contributing to the etiology of the condition [4].

SCA48 is a recently discovered rare subtype of SCAs first reported in a Spanish kindred [5], characterized by an early-onset cerebellar cognitive-affective syndrome followed by spinocerebellar ataxia. This condition presents with a spectrum of symptoms including ataxia, cognitive impairment, psychiatric dysfunction, various movement disorders encompassing hypokinetic and hyperkinetic features, and positive pyramidal tract signs which are notably complex, reflecting the heterogeneity of the disorder [6]. SCA48 is caused by the mutations in the STIP1 homology and U-box-containing protein 1 (*STUB1*) gene and showed an autosomal dominant hereditary pattern (OMIM, 607207) [5]. Variants identified in *STUB1* were initially implicated as causative mutations in an autosomal recessive spinocerebellar ataxia type 16 (SCAR16), exhibiting similar clinical symptoms with SCA48 [7]. Recent researches also have indicated a genetic synergy between *STUB1* and *ATXN8* expanded alleles, which may play a role in the pathogenic mechanisms of SCA8 [8]. Additionally, studies have pointed out an interaction between the digenic TBP/STUB1 inheritance and incomplete penetrance of SCA17. The TBP alleles with ≥ 47 repeats cause a monogenic dominant disorder SCA17, while intermediate expanded alleles (40–46) represent a digenic TBP/STUB1 disease SCA^*TBP/STUB1*^ [9, 10]. Given the resemblance in symptoms among these patients, it is possible that *STUB1* plays a key role in preserving cerebellar function.

However, the precise mechanism underlying this association remains incompletely elucidated. The *STUB1* gene encodes the C-terminus of the HSC70-interacting protein (CHIP), serving dual functions as both an E3 ubiquitin ligase and a molecular cochaperone [11, 12]. CHIP contains three domains, among these the carboxyl-terminal U-box serves as a ubiquitin ligase recruiting E2 ubiquitin-conjugating enzymes, and the N-terminal tetratricopeptide response for recruiting chaperones acting as a means of completing regulate proteostasis, these two domains are separated by a central helical hairpin region (residues 128–225) [13, 14]. Previous studies have elucidated the central role of CHIP in regulating protein quality control through its E3 ligase activity and the loss of these functions can result in protein misfolding and pathological aggregation [15].

In this study, we presented a Chinese SCA48 family involving a novel heterozygous variant (c.755 A>C) in the *STUB1* gene, which was located within the U-box domain. The patient exhibited early adult-onset multisystemic ataxia, along with complications such as hand tremors, diminished concentration, and frequent occurrence of depression or anxiety. Furthermore, we conducted in vitro functional experiments to investigate the pathogenicity of the identified mutation and provided new evidence for *STUB1*-disease as an autosomal-dominant disease.

## Methods

### Participants and clinical examination

The family was recruited from the Fujian Medical University Union Hospital. Peripheral blood was collected from three affected individuals and one asymptomatic member for genetic analysis. The diagnosis of SCA was made according to the Harding diagnostic criteria completed by two experienced neurologists. The study protocol was approved by the institutional review board of Fujian Medical University Union Hospital. All participants signed written informed consent.

### Genetic analysis

The genomic DNA of each subject was extracted from peripheral blood leukocytes using a TIANamp Genomic DNA Kit (Tiangen) according to the manufacturer’s instructions. Genomic DNA sample of the proband from leukocytes was captured using xGEN Exome research panel V2.0 Probe Set and was next proceeded to sequencing on the Illumina Hiseq sequencer (Illumina Inc., San Diego, CA) platform following the manufacturer protocols, then all of the genomic DNA samples were followed by Sanger sequencing for mutation site verfication on an ABI 3730 Genetic Analyzer (Foster City, CA, United States).

### Bioinformatic analysis

The variant was evaluated by their absence or frequency in the public single-nucleotide polymorphism database (dbSNP), 1,000 genomes, and ExAc (Exome Aggregation Consortium). The SIFT, 1 PolyPhen-22, and Mutation Taster3 were used to assess the functional effects of the missense variant. The pathogenicity analysis was conducted according to the ACMG standard.

### Literature search

We conducted a literature searched in the OMIM, PubMed and EMBASE databases up to March 2024 using the keywords “SCAR16” OR “SCA48” OR “STUB1 mutation” and summarized the relevant articles. We also searched in the Wanfang Data Knowledge Service Platform and China National Knowledge Infrastructure (CNKI) using the same keyword.

### Plasmid construction

Minigene plasmids were designed to determine the pathogenesis of *STUB1* c.755 A>C variant containing the full-length wild-type sequences of the *STUB1* gene. The genomic sequences were PCR amplified using a KOD-plus-neo kit (TOYOBO) from human DNA. Then the PCR fragments were cloned into a pCMV-cDNA-BGH-expressing plasmid using a ClonExpress MutiS One Step Cloning Kit (Vazyme Biotech). The DNA sequence of FLAG was cloned and added in the tail of the *STUB1* gene for immuno-blotting analysis and the DNA sequence of P2A-mCherry was added in the tail of the *STUB1* gene for immunofluorescence. The *STUB1* c.755 A>C variant and positive control *STUB1* c.682C>T and c.832del mutations were created by site-directed mutagenesis on the wild-type minigene plasmid using a Mut Express II Fast Mutagenesis Kit V2 (C214-01/02) according to the manufacturer’s instructions. The constructed plasmids were purfied by transformation in *E. coli* DH5a and verified by Sanger sequencing.

### PBMCs isolation

For isolation of peripheral blood mononuclear cells (PBMCs), venous blood from patients and was collected into BD Mononuclear Cell Preparation Tubes (BD). Specimens were centrifuged at 1800 × g for 20 min at room temperature (RT), the supernatant containing mononuclear cells was carefully aspirated and washed using phosphate buffered saline (PBS). Residual erythrocytes were lyzed in lysis buffer (Solarbio), cells were washed again in PBS, and pelleted at 300 × g for 10 min at RT.

### Cell culture and transfection

The HEK293T cell lines were cultured in Dulbecco’s modified Eagle’s Medium (DMEM) (Gibco), containing 10% fetal bovine serum (FBS) (Gibco) and 1% penicillin/streptomycin (PS) (Thermo Fisher Scientific). SH-SY5Y cell lines were cultured in Dulbecco’s modified Eagle’s Medium/F12 (DMEM/F12) (Gibco) containing 20% fetal bovine serum (FBS) (Gibco) and 1% penicillin/streptomycin (PS) (Thermo Fisher Scientific). Cells were dissociated using 0.05% Trypsin/EDTA (Gibco) and were cultured in a 37 °C incubator wthin a 5% CO_2_ atmosphere. All the plasmids were transfected into HEK293T cells using Lipofectamine 3000 Reagent (Thermo Fisher Scientific) and transfected into SH-SY5Y cells using FuGENE 4 K Transfection Reagent (Promega) for 48 h according to the standard instructions.

### Immunoblotting (Western blotting)

The HEK293T cells and the PBMCs were harvested in the cell lysis reagent (Sigma) supplemented with a 1% protease inhibitor PMSF (Beyotime) and centrifuged at 12,000 rpm for 20 min at 4 °C.Protein extracts were separated on 12% SDS-PAGE gels and immunoblotted with primary antibodies. HRP-conjugated secondary antibodies (1:2000, Beyotime) were used to detect primary antibodies and proteins were visualized by chemiluminescence (Meilunbio). The primary antibodies used for immunoblotting were anti-flag (1:1000, Thermo Fisher), anti-Tublin (1:1000, Abcam), anti-CHIP (1:5000 Proteintech), anti-vinculin (1:10,000 Proteintech), and anti-ubiquitin (1:1000, Abcam).

### Immunofluorescence analysis

SH-SY5Y cells cultured on glass coverslips were fixed with 4% PFA diluted in PBS for 30 min at room temperature. Then incubated with primary tau antibody (1:1,000, Genetex) followed by Alexa Fluor 488-conjugated anti-mouse secondary antibody (1:500, Thermo Fisher Scientific). The primary and secondary antibodies were both diluted in PBS containing 1% BSA and 0.3% Tx-100. At last, samples were briefly stained with DAPI (1:5000, Sigma). Images were captured with Leica TCS SP8 confocal microscopy.

## Results

### Clinical features of the patients in the SCA family

**Fig. 1:**
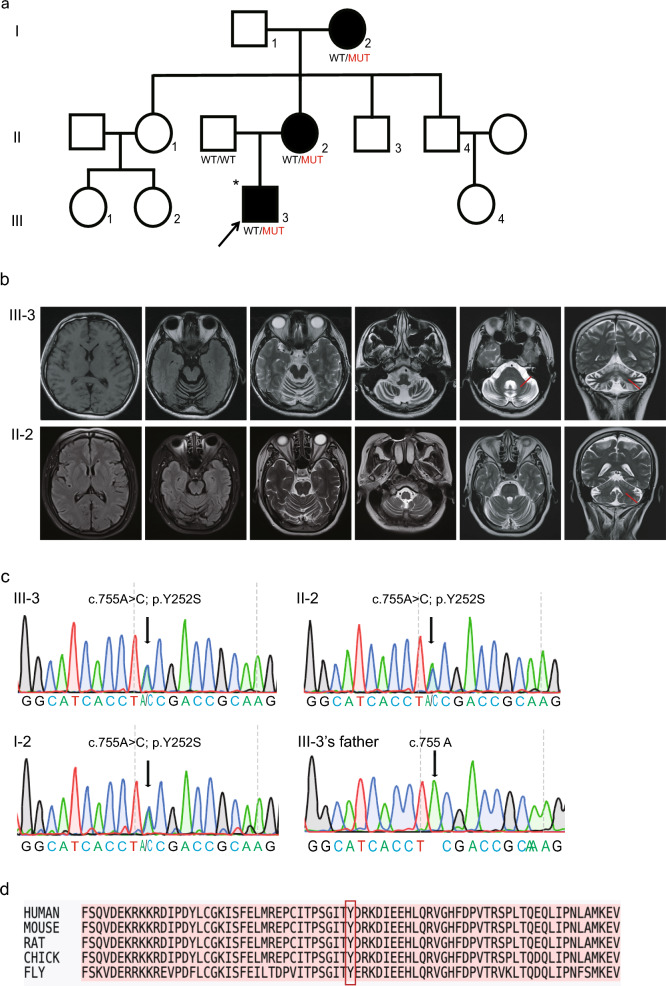
Clinical features and genetic analysis of the pedigree with *STUB1* c.755A>C variant. **a** The pedigree of the SCA48 family with the rare heterozygous missense variant, c.755A>C (p.Y252S), in the *STUB1* gene. WT/MUT = heterozygous carrier of the *STUB1* mutation; WT/WT = no mutation in *STUB1* gene; open symbols = unaffected; filled symbols = affected; the arrow indicates the proband. Asterisks indicate family members whose whole genomes were sequenced. **b** Brain MRI scans of the SCA patients (III-3 and II-2) in the family show cerebellar atrophy. **c** Sanger sequencing of the proband (III-3), his mother (II-2), his grandmother (I-2), and his father. **d** The amino acid Y252 is located within a highly conserved region of the CHIP across different species. The red arrow indicates the presence of a bilateral dentate nuclei T2weighted hyperintensity

The pedigree of the SCA family was shown in Fig. 1a. The proband (III-3) was a 24-year-old man transferred to our department in 2022. The patient presented with a 3-year history of progressively worsening gait instability. Initially, he exhibited mild unsteadiness during ambulation, accompanied by subtle dysarthria, albeit still capable of executing running and jumping maneuvers. Subsequent progression manifested 1 year ago, with a marked deterioration in gait stability rendering him incapable of motor activities. Concurrently, he developed dysarthria, dysphagia, bilateral hand tremors during working, diminished concentration, academic underperformance, heightened irritability, and frequent self-reported occurrences of depressive or anxious states. Upon neurological examination, the patient exhibited bilateral nystagmus, dysarthria, a broad-based gait, increased tone and hyperreflexia in the lower limbs, positive bilateral finger-to-nose and heel-to-shin tests, positive Romberg sign, and impaired tandem gait. At last evaluation, SARA score was 11 and the FARS-ADL score was 13. The proband’s mother (II-2) showed subtle impaired tandem gait and had a history of memory impairment when she was around 45 years old. She also showed slightly positive bilateral finger-to-nose test in neurological examination. She had lower SARA score of 5 and had minimal impact on daily life. His grandmother (I-2) had an unsteady gait history in the past 20 years (around 55 years old). Her neurological examination also revealed progressive dysarthria, hyperreflexia in the lower limbs, and a positive bilateral finger-to-nose test, while the heel-to-shin test was unable to be completed. The axial MRI imaging of the proband and his mother showed cerebellar atrophy, the coronal MRI imaging showed the dentate nuclei hyperintensity. In the imaging studies of SCA48, a specific presentation named as “crab sign” was identified which was also present in the proband’s (III-3) imaging [16] (Fig. 1b). His grandmother also had cerebral atrophy showed in her MRI report 5 years ago but the MRI image was not saved.

### Identification of a novel heterozygous variant in STUB1 gene

Considering the patient’s clinical presentation and family history, genetic testing of the proband for dynamic mutations for *ATXN1, ATXN2, ATXN3, CACNA1A, ATXN7, ATXN8OS, PPP2R2B, TBP, ATN1,* and *FXN* gene was performed. The results indicated that all the repeat expansions were within normal ranges (see Additional file 1). Whole-exome sequencing (WES) of the proband revealed a novel heterozygous variant c.755C>A (p.Y252S) in the *STUB1* gene, which was subsequently confirmed by Sanger sequencing (Fig. 1c). Sanger sequencing confirmed the presence of the *STUB1* c.755 C>A variant in both the proband’s mother and grandmother (Fig. 1c) but not in the proband’s father.

### Prediction of pathogenicity of the STUB1 variant by bioinformatics analysis

The *STUB1* c.755 C>A (p.Y252S) variant was interpreted as a variant of uncertain significance (VUS) according to the 2019 American College of Medical Genetics and Genomics (ACMG) guidelines [17]. The variant was absent from the population databases 1000 Genomes and ExAC, as well as from 1,000 ethnicity-matched controls (PM2_Supporting). Silico analyses predicted the novel variant c.755 C>A to be “damaging” and “probably damaging” with SIFT and PolyPhen-2, respectively. Additionally, the variant was predicted to be “disease-causing” with MutationTaster, “Deleterious” with LRT, and other predictions of the PROVEAN, VEST3, M-CAP, and CADD are all “Damaging” (PP3). All the in-silico predictions were listed in Table 1. A sequence alignment also demonstrates the evolutionary conservation of Y252 in the U-Box domain of CHIP across the indicated species (Fig. 1d). Through the analysis of the hereditary patterns within this family, we observed that the proband, along with the mother and grandmother, all exhibited the disease phenotype and carried the c.755C>A (p.Y252S) mutation in the *STUB1* gene. In contrast, the unaffected father didn’t carry this mutation, indicating a pattern consistent with familial co-segregation.

**Table 1:** In silico prediction of the *STUB1* c.755 C>A variant

Algorithm	Score	Predictiont
SIFT	0.0	Damaging
Polyphen-2_HDIV	1.0	Probably_damaging
Polyphen-2_HVAR	1.0	Probably_damaging
LRT	0.000	Deleterious
MutationTaster	1	Disease_causing
MutationAssessor	4.385	High
PROVEAN	− 7.94	Damaging
VEST3	0.964	Damaging
M-CAP	0.224	Damaging
CADD	24.8	Damaging

### Phenotypes of STUB1 mutations

By searching various English and Chinese databases for literature related to the *STUB1* mutation, we found that 13 papers were related to SCA48 [5, 18–29], 15 papers were associated with SCAR16 [7, 15, 24, 30–41], and 8 papers were related to SCA^*TBP/STUB1*^ [9, 10, 23, 28, 42–45]. Given the same mutated gene *STUB1* and potential shared pathogenic mechanisms among these diseases, we compared the clinical phenotypes of these diseases in the published literatures. Forty patients with SCAR16 and 261 patients with SCA48 were caused by *STUB1* gene mutations, and 97 patients with SCA^*TBP/STUB1*^ were associated with *STUB1* gene mutations, which were summarized in Table 2 according to the clinical characteristics. SCA48 had the largest reported population (261) and SCA^*TBP/STUB1*^ had the latest onset age (44), while SCAR16 had the widest range of onset age (5–76), which might be due to the different inheritance modes. As for the clinical symptoms, all patients either with SCAR16 or with SCA48 exhibited cerebellar features, which were the most common characteristics observed. SCA48 had a higher percentage of patients with movement disorders and psychiatric disorders but a lower percentage of epilepsy and hypogonadism. Additionally, more than half of the patients with SCA48 presented with cognitive impairment in the reported cases. Patients with SCAR16 exhibits a slightly higher incidence of hyperreflexia and urinary system symptoms. In the reported cases, all SCAR16 patients showed cerebellar atrophy while 4% of SCA48 patients did not show cerebellar atrophy on CT or MRI images. Apart from the different inheritance patterns, the clinical features of SCA16 and SCA48 were almost similar and there were no distinct symptoms to different one from another. Regarding the SCA^*TBP/STUB1*^, we found that apart from more common cognitive impairment and movement disorders, the prevalence of other symptoms did not significantly differ from those observed in the SCA48, which may due to the *TBP* duplication. Thus, a larger population of cases was needed to better identify the clinical phenotypes and genotypes of SCAR16, SCA48 and SCA^*TBP/STUB1*^.

**Table 2:** Comparison of clinical phenotypes between SCAR16, SCA48 and SCA^*TBP/STUB1*^

	SCAR16 (n = 40)	SCA48 (n = 261)	SCA^*TBP/STUB1*^ (n = 97)
Onset age (mean)	22.35 (14–45)	38.00 (5–76)	44.00 (19–66)
Cerebellar features	100%	100%	100%
Cognitive impairment	23 (58%)	167 (64%)	93 (96%)
Movement disorders^†^	14 (35%)	134 (51%)	65 (67%)
Psychiatric disorders	3 (8%)	103 (39%)	42 (43%)
Hyperreflexia	12 (30%)	40 (15%)	11(11%)
Epilepsy	4 (10%)	7(3%)	2 (2%)
Hypogonadism	5 (13%)	1(0%)	0
Urinary tract symptoms	7 (18%)	14 (5%)	0
Cerebellar atrophy (CT or MRI)	38/38^‡^	48/50	22/22

^†^Movement disorders including parkinsonism, chorea, dystonia, tremor

^‡^The denominator is specified only if different from the number of total cases

### The CHIP p.Y252S abolishes ubiquitin ligase activity

**Fig. 2:**
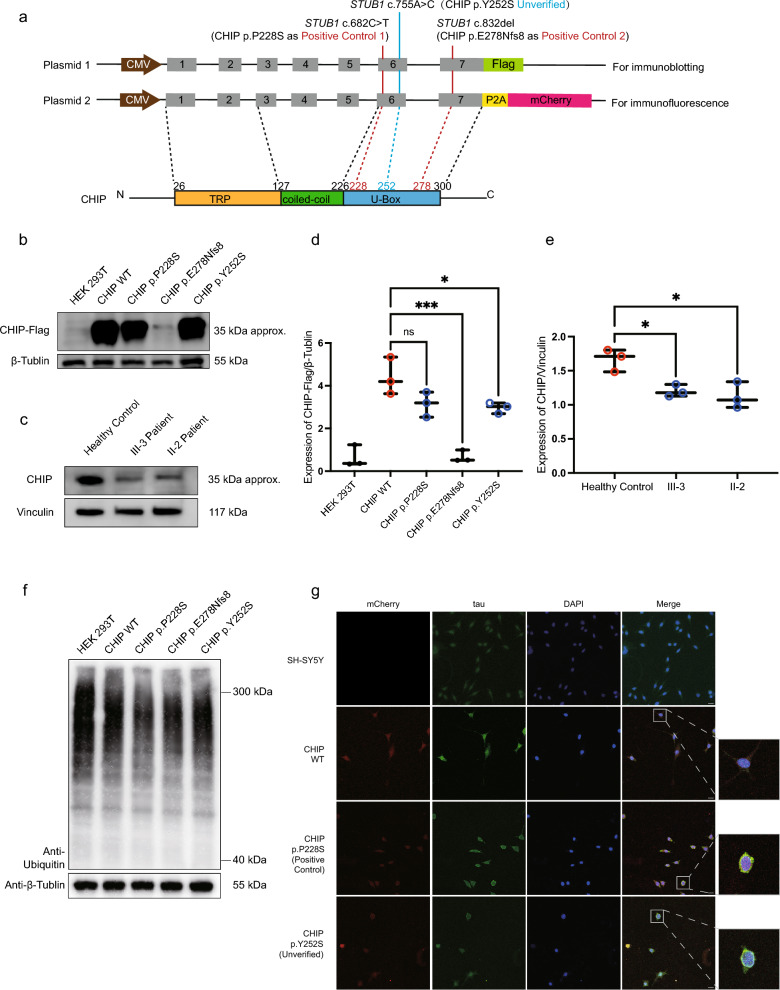
Functional characterization of the *STUB1* c.755A>C variant. **a** Schematic diagram of the minigene plasmids. Plasmid 1 is for immunoblotting analysis and Plamid 2 is for immunofluorescence. The minigene plasmids contain CMV promotor, consecutive genomic DNA of *STUB1* gene, and Flag or P2A-mCherry sequences, including *STUB1* c.755A>C, c.682 C>T or c.832del mutation in the corresponding positions respectively. Grey boxes indicate exons, and corresponding domains of CHIP are showed below. **b** Levels of CHIP-FLAG in HEK 293 T without or with mutation were quantified by performing Western Blot analysis using anti-Flag antibody, Strong bands were visible at the location of predicted molecular weight of 35 kDa except CHIP wildtype and p.E278Nfs8. Equal protein loading is demonstrated using anti-β-Tublin antibody. **c** Levels of CHIP in PBMCs from patients and healthy control were quantified by performing Western Blot analysis using anti-Flag antibody, Strong bands were visible at the location of predicted molecular weight of 35 kDa. Equal protein loading is demonstrated using anti-Vinculin antibody. **d** and **e** The statistical results of figure b and c showed the levels of CHIP expression in each group relativing to β-Tublin or Vinculin decreased. Differences in means were assessed with one-way ANOVA. Means and standard deviations are shown. n.s., non-significant (*p* > 0.05), *p < 0.05, ***p < 0.001. **d** Levels of ubiquitin modification were quantified using Western Blot analysis, Equal protein loading is demonstrated using anti-β-Tublin antibody. **e** Confocal images of SH-SY5Y cells transfected with CHIP Wildtype, p. P228S, or p. Y252S, respectively. The crop pictures show a single cell without tau aggregation (CHIP Wildtype) or with significant tau aggregation (CHIP p. P228S or p. Y252S). Tau was visualized by anti-tau antibody followed by Alexa Fluor 488-conjugated anti-mouse secondary antibody. Scale bar, 100 μm

CHIP-mediated ubiquitination protein modification requires sequential enzymatic activity involving E1 activating, E2 conjugating, and E3 ligating enzymes. The U-box domain of CHIP facilitates the binding of both E2 conjugates and substrates, thereby promoting ubiquitination modification of protein [46]. To assess ubiquitin ligase activity, we constructed *STUB1* minigene plasmids containing either wild-type sequences (named CHIP WT) or variant sequences, one with the c.755 C>A (named CHIP p. Y252S) and the other two with known pathogenic mutations (named CHIP p.P228S and CHIP p. E278Nfs8 respectively) (Fig. 2a). Notably, the total CHIP abundance of CHIP p. Y252S was significantly reduced in HEK293T cells transfected with minigene plasmids, which was consistent with the results of PBMCs derived from patients with CHIP p. Y252S (Fig. 2b–e). HEK293T cells transfected with different minigene plasmids were examined for total ubiquitin modification of proteins. HEK293T cells transfected with CHIP p. Y252S demonstrated decreased overall ubiquitination levels, akin to the positive controls CHIP p. E278Nfs8 previously validated to have impaired ubiquitin ligase activity (Fig. 2f). These findings collectively suggested that the CHIP p. Y252S variant may influence the stability of the CHIP and impair its E3 ubiquitin ligase activity.

### The CHIP p.Y252S triggers tau aggregation

Previous studies have shown that CHIP deficiency may induce tau aggregation [47]. To further elucidate the variant’s pathogenicity, we investigated the relationship between CHIP p.Y252S variant and tau aggregation. We conducted immunofluorescent staining in SH-SY5Y cells after transfecting with *STUB1* minigene plasmids expressing mCherry tag (Fig. 2a). Immunofluorescent staining demonstrated significant tau-EGFP aggregation in the cytoplasm upon overexpression of CHIP p. Y252S and the positive control CHIP p.P228S but not in CHIP WT (Fig. 2g). These findings suggested that the CHIP p.Y252S variant could lead to tau aggregation in the cytoplasm.

## Discussion

In this study, we identified a novel *STUB1* c.755 C>A mutation in a Chinese SCA48 pedigree. The proband exhibited a characterized phenotype of ataxia, dysarthria, dysphagia, tremors of hands, and cognitive impairment. Brain MRI demonstrated obvious hemispheric cerebellar atrophy and T2 dentate hyperintensities. The proband’s mother and grandmother also showed mild features of ataxia and cerebellar atrophy. The three patients in the family all carried the heterozygous *STUB1* c.755 A>C variant, which was not present in the proband’s unaffected father, suggesting co-segregation of the variant within the family. The carrier of *STUB1* mutation existed the susceptibility of affecting SCAR16, SCA48, SCA8, SCA17 and SCA^*TBP/STUB1*^. However, the SCAR16 displayed an autosomal recessive inheritance pattern which was different from the autosomal dominant inheritance pattern in this family. The repeats in the CAG/TAG of the ATXN8OS gene was also within the normal range (21/32) and the CAG/CAA repeats in the TBP was also within the normal range (34/37), which could rule out the SCA8, SCA17 and SCA^TBP/STUB1^.

SCA48, the most recently described autosomal dominant spinocerebellar ataxia due to heterozygous mutations in *STUB1* gene [6]. The reported phenotypes of SCA48 cases indicated not only classical ataxia and cognitive/behavioral dysfunction but also a wide spectrum of movement disorders. To be noticed, the SCA48 had a higher percentage of cognitive impairment and psychiatric disorders and a lower percentage of features like epilepsy and hypogonadism (Table 2). The proband we reported here showed classical ataxia features, accompanied by tremors of hands, diminished concentration, heightened irritability, and frequent depression or anxiety, consistent with the common symptoms of SCA48. Interestingly, the proband’s age of onset (21-year-old) was earlier than the average age reported in the previous literatures, which was about 35 years old. What’s more, the other two SCA patients in the family showed rather later age of onset and relatively milder phenotypes compared to the other reported cases so far which might be due to the patient’s gender or genetic factors. It needed further clinical data from following up with this family and investigation of more SCA48 cases to better understand the complex clinical phenotypes of this disease. Recently, a debate has emerged regarding the classification of SCA48 as a monogenic disorder, particularly considering the existence of a digenic form of SCA17 [9, 10, 27]. The reported case presenting with the full SCA48 clinical phenotypes and normal TBP alleles, which supporting the existence of monogenic spinocerebellar ataxia 48. This challenges the STUB1/TBP digenic model and aligns with the perspective of Barbier et al. [28].

Online in silico programs predict the STUB1 c.755 C>A (p.Y252S) variant to be deleterious. Further experiments revealed an obvious decrease in the level of CHIP with the p. Y252S mutation. The low expression level of CHIP was a confirmed feature of SCA48 [48]. Our study also revealed that the CHIP p. Y252S mutation impairs the overall ubiquitination modification and leads to tau aggregation in the cytoplasm. We supposed that the ubiquitin-modified impairment of the CHIP protein through amino acid substitution of the U-box domain which responded to the ubiquitin ligase activity [15]. The mechanisms underlying the degradation of CHIP protein required further investigation, and we speculated that it might be related to alterations in the spatial conformation. The decrease of both quality and quantity contributed to the downregulation of the ubiquitin-modified function of the CHIP and then potentially led to the aggregation of other misfolded proteins like the tau protein. It has been documented that the ubiquitination level was closely associated with the progression of neurodegenerative disorders, including the SCAs [46]. Additionally, our findings highlight the crucial role of protein aggregation clearance, such as tau aggregation, which was increased in post-mortem brain tissues of SCA48 patients, in the progression of SCA48 [24]. These findings offered evidence that tau might play an important role in SCA48 disease progression and were consistent with previous literatures [26].

## Conclusions

We identified a novel STUB1 c.755 C>A mutation in a Chinese SCA48 pedigree. We also revealed that the CHIP p. Y252S mutation impairs the overall ubiquitination modification and leads to tau aggregation in the cytoplasm. Our findings not only broaden the mutation spectrums of the SCA48 but also expand the understanding of the pathogenesis associated with *STUB1* gene mutations.

## Supplementary Information

Additional file1 (DOCX 633 KB)

## Data Availability

The WES data were deposited into the Sequence Read Archive (SRV) database under accession number PRJNA1170204 and are available at the following URL: https://www.ncbi.nlm.nih.gov/sra/PRJNA1170204

## Abbreviations

ACMG: American College of Medical Genetics and Genomics
CHIP: C-terminus of HSC70-interacting protein
CNKI: China National Knowledge Infrastructure
dbSNP: Single-nucleotide polymorphism database
ExAc: Exome Aggregation Consortium
PBMC: Peripheral blood mononuclear cell
SCA48: Autosomal dominant spinocerebellar ataxia type 48
SCAR16: Autosomal recessive spinocerebellar ataxia type 16
SCA17-DI: Spinocerebellar ataxia type 17-digenic TBP/STUB1 disease
SCAs: Spinocerebellar ataxias
STUB1: STIP1 homology and U-box-containing protein 1
TBP: TATA-binding protein
VUS: Variant of uncertain significance
WES: Whole-exome sequencing
