# Supplementary material for: Clinical and functional characterization of a novel STUB1 mutation in a Chinese spinocerebellar ataxia 48 pedigree
Source: Orphanet J Rare Dis. 2024 Dec 20;19:471. doi: 10.1186/s13023-024-03456-8 (PMC11662494; doi:10.1186/s13023-024-03456-8)
Supplement: Supplementary file 1 — Additional file1 (DOCX 633 KB) [file 13023_2024_3456_MOESM1_ESM.docx]

## **Additional files 1**

## **the Fluorescent PCR-capillary Electrophoresis Results in Related Genes**


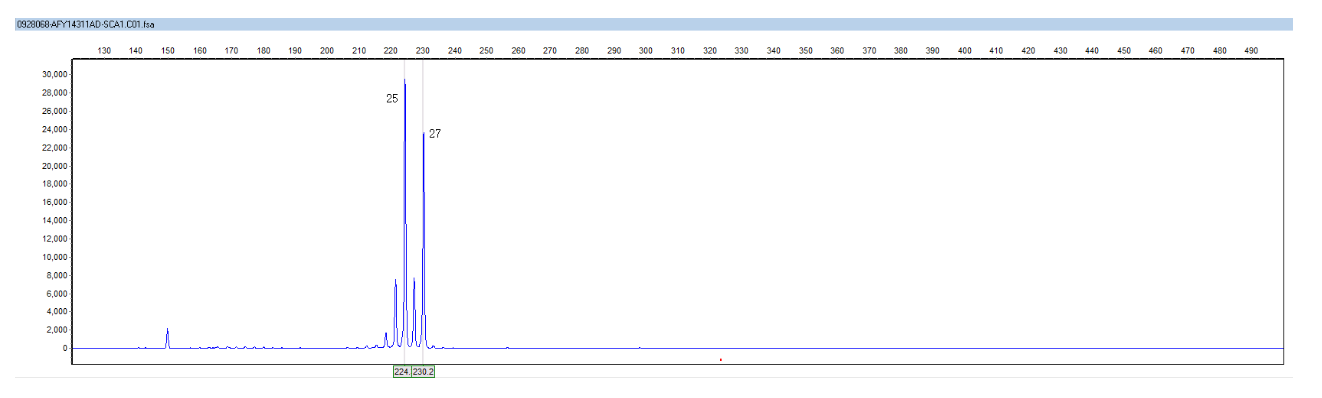


The number of repeats in the three-base repeat region (CAG) n of the two alleles of the ATXN1 gene in the subjects was 25/27.


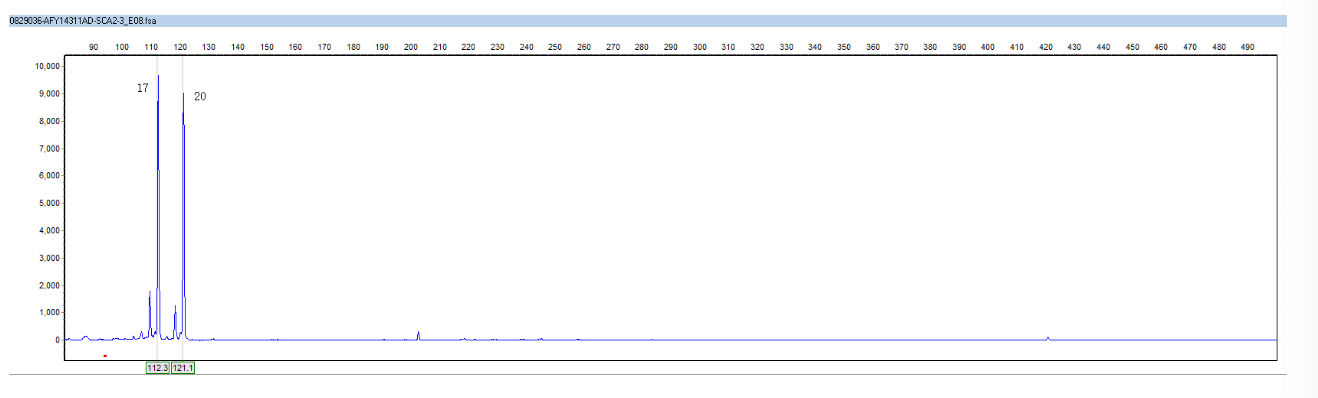


The number of repeats in the three-base repeat region (CAG) n of the two alleles of the ATXN2 gene in the subjects was 17/20.


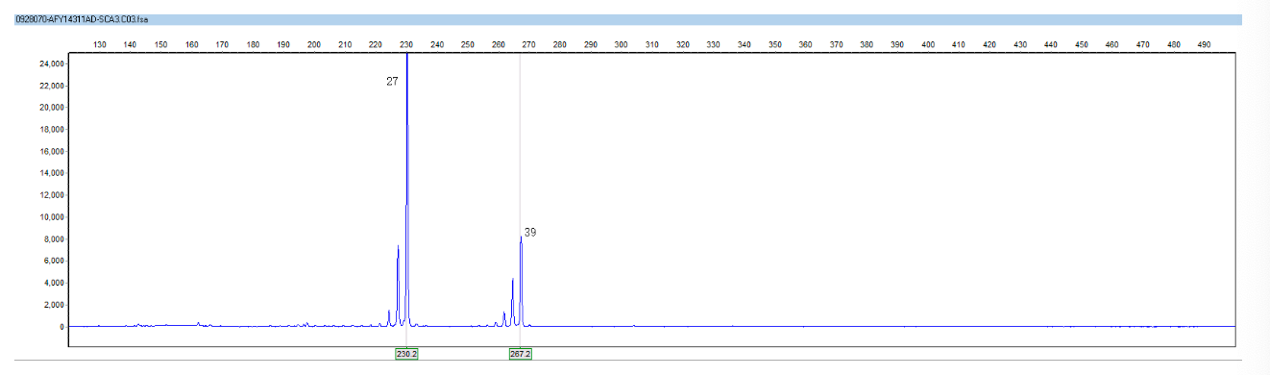


The number of repeats in the three-base repeat region (CAG) n of the two alleles of the ATXN3 gene in the subjects was 27/39.


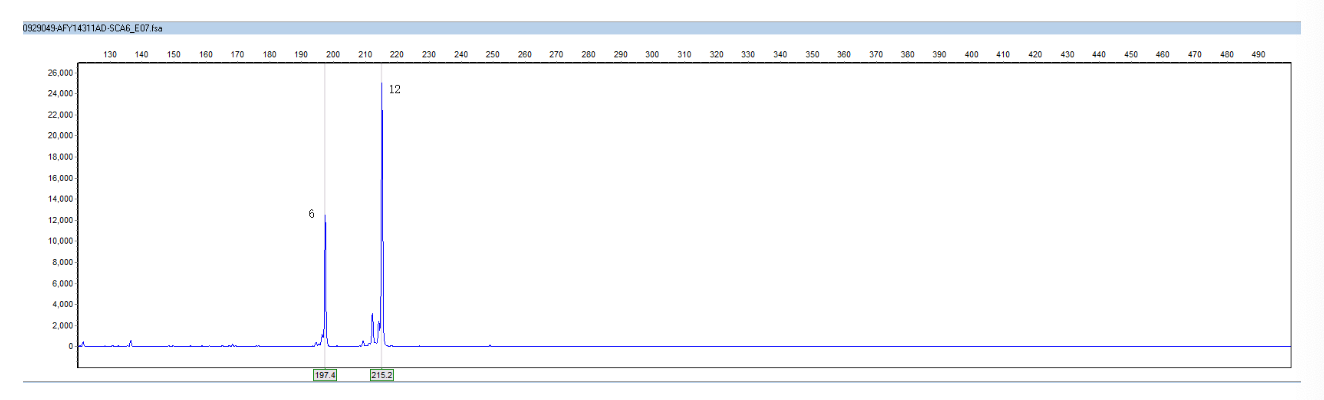


The number of repeats in the three-base repeat region (CAG) n of the two alleles of the CACNA1A gene in the subjects was 6/12.


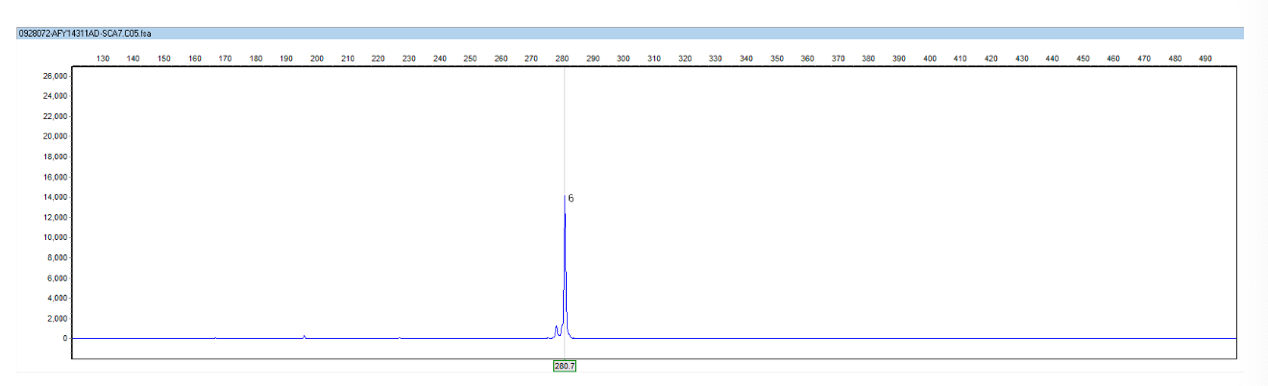


The number of repeats in the three-base repeat region (CAG) n of the two alleles of the ATXN7 gene in the subjects was 6/6.


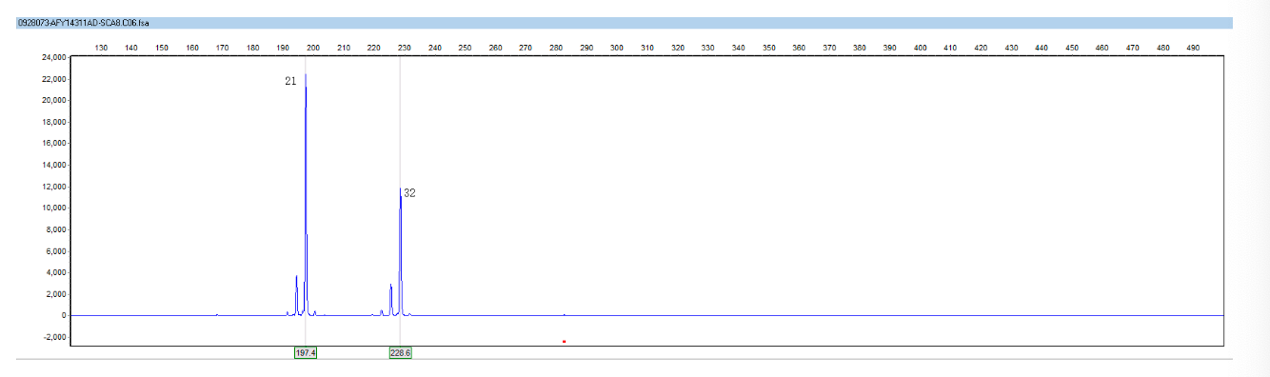


The number of repeats in the three-base repeat region (CAG/TAG) n of the two alleles of the ATXN8OS gene in the subjects was 21/32.


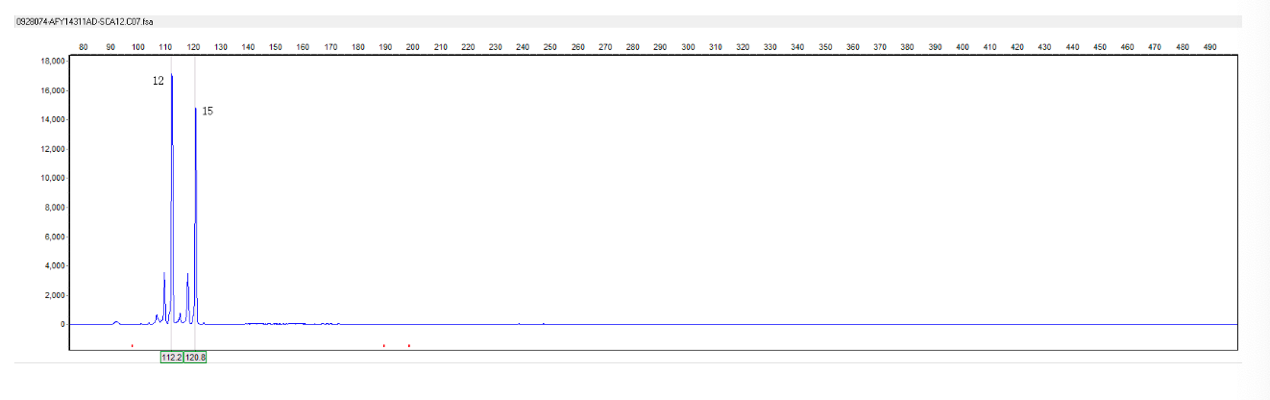


The number of repeats in the three-base repeat region (CAG) n of the two alleles of the PPP2R2B gene in the subjects was 12/15.


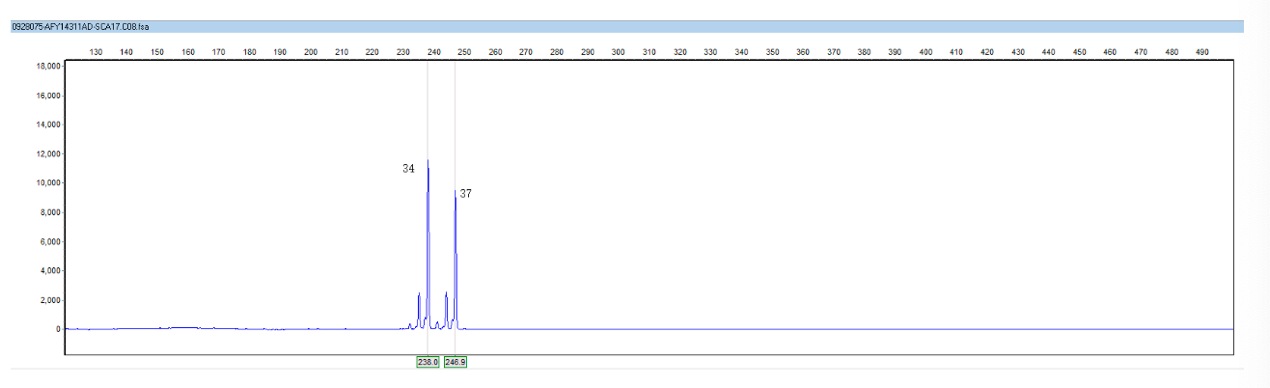


The number of repeats in the three-base repeat region (CAG/CAA) n of the two alleles of the TBP gene in the subjects was 34/37.


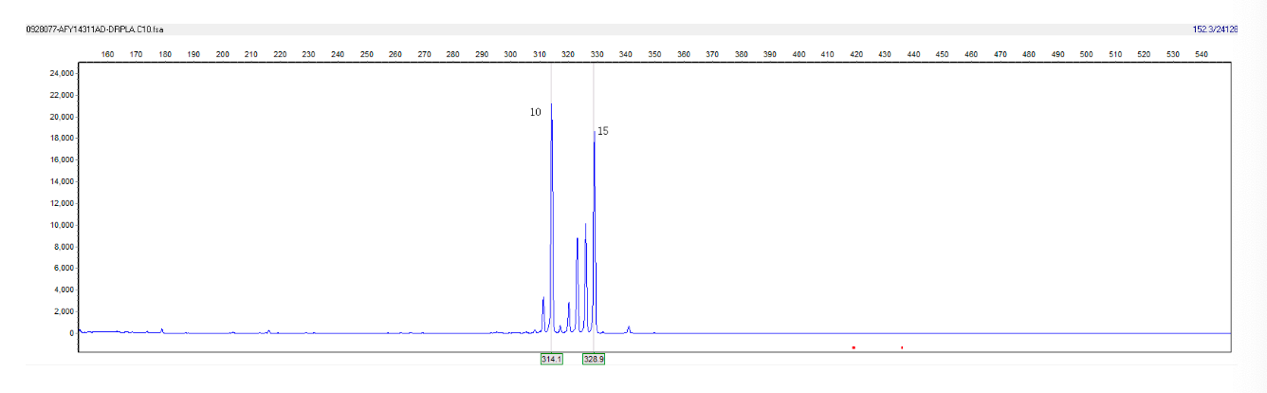


The number of repeats in the three-base repeat region (CAG) n of the two alleles of the ATN1 gene in the subjects was 10/15.


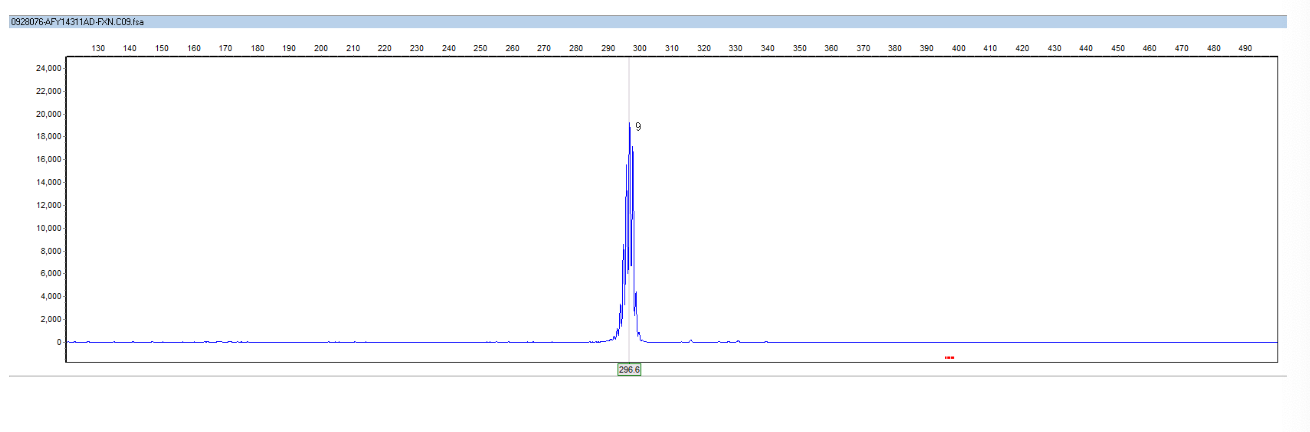


The number of repeats in the three-base repeat region (GAA)n of the two alleles of the FXN gene in the subjects was 9/9.

## **Summary of ataxia related 10 types of dynamic mutation detection**

| Disease | Gene | Repetition |
| --- | --- | --- |
| SCA1 | ATXN1 | 25/27 |
| SCA2 | ATXN2 | 17/20 |
| SCA3 | ATXN3 | 27/39 |
| SCA6 | CACNA1A | 6/12 |
| SCA7 | ATXN7 | 6/6 |
| SCA8 | ATXN8OS | 21/32 |
| SCA12 | PPP2R2B | 12/15 |
| SCA17 | TBP | 34/37 |
| DRPLA | ATN1 | 10/15 |
| FRDA | FXN | 9/9 |
